# Differences in the genomic diversity, structure, and inbreeding patterns in wild and managed populations of *Agave potatorum* Zucc. used in the production of Tobalá mezcal in Southern Mexico

**DOI:** 10.1371/journal.pone.0294534

**Published:** 2023-11-16

**Authors:** Karen Y. Ruiz Mondragón, Anastasia Klimova, Erika Aguirre-Planter, Alfonso Valiente-Banuet, Rafael Lira, Guillermo Sanchez-de la Vega, Luis E. Eguiarte

**Affiliations:** 1 Departamento de Ecología Evolutiva, Instituto de Ecología, Universidad Nacional Autónoma de México, Ciudad de México, México; 2 Departamento de Ecología de la Biodiversidad, Instituto de Ecología, Universidad Nacional Autónoma de México, Ciudad de México, México; 3 Centro de Ciencias de la Complejidad, Universidad Nacional Autónoma de México, Ciudad Universitaria, Ciudad de México, México; 4 Laboratorio de Recursos Naturales, Unidad de Biotecnología y Prototipos (UBIPRO), Facultad de Estudios Superiores Iztacala, Universidad Nacional Autónoma de México, Tlalnepantla de Baz, Estado de México, México; Institute for Biological Research, University of Belgrade, SERBIA

## Abstract

*Agave potatorum* Zucc. locally known as Tobalá, is an important species for mezcal production. It is a perennial species that takes 10 to 15 years to reach reproductive age. Because of high demand of Tobalá mezcal and the slow maturation of the plants, its wild populations have been under intense anthropogenic pressure. The main objective of this study was to estimate the genome-wide diversity in *A*. *potatorum* and determine if the type of management has had any effect on its diversity, inbreeding and structure. We analyzed 174 individuals (105 wild, 42 cultivated and 27 from nurseries) from 34 sites with a reduced representation genomic method (ddRADseq), using 14,875 SNPs. The diversity measured as expected heterozygosity was higher in the nursery and wild plants than in cultivated samples. We did not find private alleles in the cultivated and nursery plants, which indicates that the individuals under management recently derived from wild populations, which was supported by higher gene flow estimated from wild populations to the managed plants. We found low but positive levels of inbreeding (*F*_*IS*_ = 0.082), probably related to isolation of the populations. We detected low genetic differentiation among populations (*F*_*ST*_ = 0.0796), with positive and significant isolation by distance. The population genetic structure in the species seems to be related to elevation and ecology, with higher gene flow among populations in less fragmented areas. We detected an outlier locus related to the recognition of pollen, which is also relevant to self-incompatibility protein (SI). Due to seed harvest and long generation time, the loss of diversity in *A*. *potatorum* has been gradual and artificial selection and incipient management have not yet caused drastic differences between cultivated and wild plants. Also, we described an agroecological alternative to the uncontrolled extraction of wild individuals.

## Introduction

*Agave* L., is a species-rich genus, with over 200 described species native to America’s arid lands [1–4]. Most species occur in Mexico, with many of them being endemics and microendemics [1, 3, 5]. Although *Agave* species have had enormous cultural and economic importance, currently in Mexico, the primary use of these plants is for the production of alcoholic beverages such as tequila and mezcal [2, 6, 7].

Among the alcoholic beverages production in Mexico, the tequila and mezcal industry is the second most important economic activity. Over 50% of this production is destined for export [8], which in the year 2018 represented over 53 million USD of revenue [8]. Nevertheless, to produce mezcal, large quantities of raw materials (i.e., *Agave* mature plants, water, and firewood) are required. The ever-growing demand and high requirements for raw materials have resulted in a considerable increase in land use change, with lands destined for *Agave* plant cultivation increasing from 8,663 hectares in 2003 to 21,878 ha in 2017 [8, 9].

Currently, mezcal production is considered unsustainable [10]. This is because only a few *Agave* species are cultivated in commercial monoculture plantations (i.e., *A*. *tequilana* Weber for tequila or *A*. *angustifolia* Haw. for Espadín mezcal production) [7, 11]. The recent increase in tequila and Espadín production has resulted in important ecological problems, including the removal of native vegetation, forest clearing, substitution of traditional crops for *Agave* planting, use of agrochemicals, soil contamination, and erosion. In contrast, other *Agave* species used in mezcal production are mainly extracted from the wild, resulting in overexploitation of natural populations [2, 10, 12–15]. However, as the mezcal industry keeps growing, several species of *Agave* have begun to be cultivated (i.e., *A*. *potatorum*, *A*. *marmorata* Roezl., *A*. *karwinskii* Zucc., *A*. *cupreata* Trel. & A. Berger) with seedlings derived from wild, nurseries or through clonal propagation. Furthermore, both wild and cultivated *Agave* specimens used in the production of mezcal or tequila are harvested *prior* to blossom, when the plants concentrate all their sugars and nutrients before producing their inflorescence, as these resources are used to produce tequila or mezcal. The removal of the inflorescences has consequences, it not only prevents the agave plants from producing nectar, pollen or seeds, but also impacts the populations of pollinators, causing a decline in the natural recruitment and a reduction in the genetic variation [14–18].

The increasing demand for mezcal may be already generating an “extinction debt” [19], as some *Agave* species have had a greater demand due to the unique organoleptic properties such as *A*. *potatorum*, known as Tobalá [16, 20].

*Agave potatorum* is an endemic species, restricted to the Sierra Madre del Sur, Tehuacán Valley in the state of Puebla, and part of the state of Oaxaca mountains, in Mexico [20]. It is found in evergreen sclerophyllous vegetation named Mexical [21], and in the oak and pine forest grassy slopes at an elevation between 1,760 to 2,300 m [2, 18]. It is a semelparous, monocarpic species that grows slowly (compared to *A*. *tequilana* and A. *angustifolia*, for instance). It takes 10 to 15 years to flower, blooming from August to November. The fruits and seeds mature from November to March. The reproductive individuals are of relatively small size compared to other Agaves that produce mezcal [20, 22]. Sexual reproduction is the primary reproductive strategy of this species; however, while rare, some vegetative propagation has been reported in disturbed populations [23]. Each plant can produce 2,000 to 9,500 seeds [23]. *Agave potatorum* depends mainly on bats for pollination, particularly the lesser long-nosed bat (*Leptonycteris yerbabuenae* Martínez & Villa, 1940); also other bats and animals can also be involved [22, 24]. In populations characterized by a high density of flowering *Agave* species, nectarivorous bats exhibit greater foraging and pollination efficiency, due to their increased flower visitation rates, favored by the continuous nectar reward [22].

As a result of the growing demand for mezcal, high extraction rates and slow maturation, *A*. *potatorum* has become one of the most threatened *Agave* species [10], with IUCN Red List recognizing that populations are decreasing [25]. For instance, it was estimated that near San Luis Atolotitlán (SLA), Puebla, Mexico, 54% - 87% of the wild mature plants are extracted annually [26]. Furthermore, as this *Agave* is smaller than, for example, *A*. *angustifolia*, more individuals are needed to produce the same amount of mezcal [13, 14]. Due to concerns regarding the decline of wild populations, *in situ* sustainable agro-management has been proposed and is currently underway in two villages: San Luis Atolotitlán (SLA) and San Juan Raya (SJR), both in the state of Puebla in Central Mexico. In collaboration with researchers, producers have implemented collective nurseries to supply the demand of seeds and seedlings and avoid extracting wild individuals [26]. According to Delgado-Lemus [13], the cultivation of this species entails an incipient domestication process [10, 13].

Domestication is a complex evolutionary process in which human selection leads to morphological and physiological changes in plants, resulting in the modification of the genotypes and phenotypes and the differentiation of the domesticated varieties from their wild ancestors [27]. As a result of the demographic process and artificial selection, a reduction in the crop’s genetic diversity through the process known as “domestication bottleneck” has been observed in many crop species [27–29]. Therefore, morphological, physiological, and genetic changes associated with domestication have been extensively studied [30–32]. However, most studies have focused on annual species, such as maize, rice, wheat, sorghum, and pumpkins [27, 33], whereas the domestication process in long-lived-perennials plants, such as *Agave*, grapes, palms, and tree nuts has received less attention [27]. Due to a prolonged juvenile period and sexual reproduction, the domestication of most perennial plants usually entails clonal propagation. This may imply an increase in diversity through the accumulation of somatic mutations in already heterozygous genotypes [27, 34, 35].

Recently several studies have focused on domestication as a model for understanding the evolutionary processes [31, 36–38]. Under this model and given the ecological, cultural, and economic importance of *A*. *potatorum*, we consider it is vital to understand how the management of this species is carried out in its distribution area (in the states of Oaxaca and Puebla, Mexico). Due to the conservation concerns, we employed a reduced representation genomic method, RADseq, to study the genetic variation in plants under different types of management. Our primary focus was to determine whether there was a loss of genetic diversity resulting from bottlenecks associated to the incipient domestication process, and artificial selection. To achieve this, we analyzed *A*. *potatorum* under different management types: wild (with no management at all), cultivated (with management), and nursery (produced in a shade house).

We believe it is crucial to understand the genetic similarities and differences between cultivated and wild plants, as wild plants can potentially serve as germplasm reservoirs for future crop improvements. Additionally, we conducted an exploratory analysis of outlier loci, as we wanted to identify agronomically or ecologically important alleles in wild populations. In cultivated localities our goal was to detect loci associated with the production of secondary metabolites that contribute to the perceived quality of this mezcal.

Given the incipient cultivation practices, we anticipated finding higher genetic diversity in undisturbed wild populations, with still low genetic differentiation from cultivated populations, and few unique alleles in managed localities. Furthermore, we expected to find evidence of gene flow between wild and cultivated plants. Additionally, we presumed that nursery and cultivated plants would exhibit closer genetic relationships, accompanied by higher levels of inbreeding.

## Materials and methods

### Plant material

**Fig 1:**
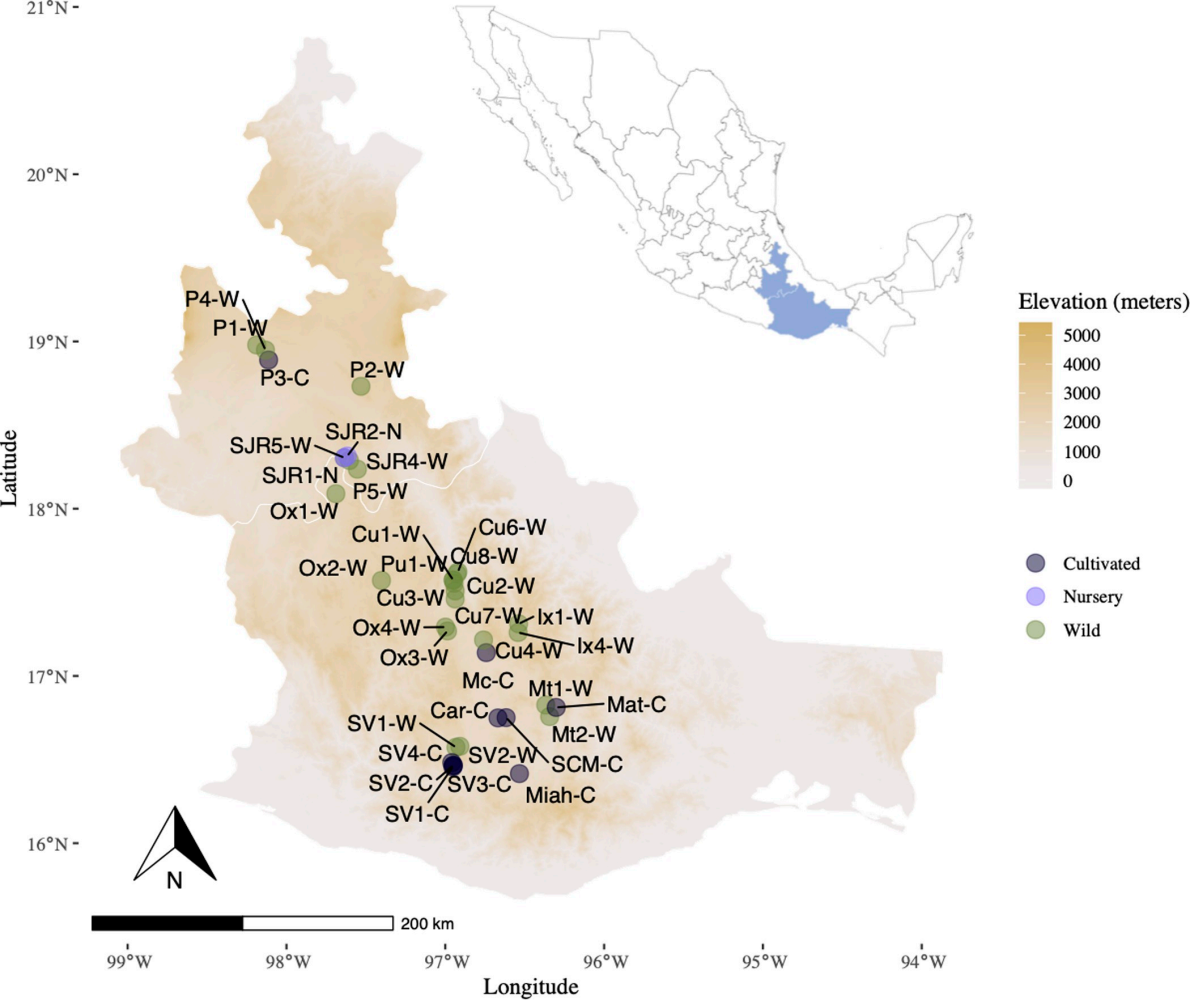
Localities of *Agave potatorum* in Puebla and Oaxaca, Mexico. Colors shading on the map show the elevation in the collection area, and the colored dots correspond to different types of management: wild (green), cultivated (dark purple), and nursery (purple).

We collected leaves from 192 specimens of *Agave potatorum* (S1 Table in S1 File and Fig 1) covering its entire distribution range in the states of Puebla and Oaxaca, Mexico. The distribution of the sampling sites (Fig 1) was plotted using R packages *raster*, *elevatr*, *ggspatial* and *ggplot2*. A total of 34 sampling localities were visited, including different management types: wild (22 sampling sites), two nursery—sites specially dedicated to the production of seedlings—(SJR1-N and SJR2-N) and cultivated (10 sampling sites) with approximately 20 years of cultivation. In most cases cultivated plants were collected at polycultures, alternated with *A*. *angustifolia* or *A*. *karwinskii*. We only found one monoculture of *A*. *potatorum* in Puebla (P3-C). Apparently, due to the incipient domestication of *A*. *potatorum*, the number of cultivation sites (such as plantations or nurseries) for this species is still limited.

The sampled plants had different ages, the wild and cultivated plants were categorized as adults, and the nursery populations were young individuals (less than two years old). One nursery locality was in a recent set-up greenhouse, and the second one (SJR1-N) came from a shade house, where seeds have been growing for eight years; the mother plants come from a plantation under agroecological management in Cerro los Pesos, near Zapotitlán, both in San Juan Raya, Puebla (S1 Table in S1 File and Fig 1). All samples were collected and stored until DNA extraction at -80°C.

### DNA extraction and sequencing

Genomic DNA was extracted from leaf tissue using a modified "Mini-Prep" CTAB protocol [39]. For the exact modifications of the protocol, see [11]. DNA quantity and quality were first examined using a 1% agarose electrophoresis gel. Then, samples of adequate quality were quantified using a Qubit 3.0 fluorometer and Qubit dsDNA broad-range kit. Library preparation for RADseq and sequencing were performed at the Biotechnology Center of the University of Wisconsin-Madison (https://biotech.wisc.edu/). Each sample was digested using two methylation-sensitive restriction enzymes (*PstI* and *MspI*). Samples were pooled in equimolar concentration after specific barcodes were ligated to them and sequenced using the Illumina NovaSeq 2x150 platform (Illumina, Inc., San Diego, CA, United States).

### SNP calling and filtering

Demultiplexing of the samples, initial data filtering and SNP calling were performed with Ipyrad v.0.9.77 software [40], using the ddRAD (double digestion RAD) method analysis strategy. The transcriptome of *A*. *tequilana* (GAHU00000000.1; [41]) was used as a reference. Filtering of the vcf file was done using VCFtools v.0.1.15 [42]. To ensure data quality, we performed several filters retaining only bi-allelic sites, with a mean minimum depth of over 12, and no InDels. We removed SNPs with a significant deviation from Hardy-Weinberg equilibrium (function—hwe 1e-07), with a minor allele frequency (MAF) of <0.05, and individuals with more than 80% missing data. Using PLINK v.1.9 software [43], we excluded loci with high linkage disequilibrium (LD); thus, loci presenting *r*^*2*^ of over 0.2 were removed from the further analysis.

### Genetic diversity

The number of private alleles (*PA*) and multilocus genotypes (*mlg*) were calculated with the *poppr* R package [44]. Additionally, we estimated the following genomic diversity statistics: the overall heterozygosity (*H*_*T*_), observed heterozygosity (*H*_*O*_), and expected heterozygosity (*H*_*E*_) per locus, in three data sets: 1) per each sampling site; 2) for each management type (wild, cultivated and nursery) and 3) for each reproductive age (adults *vs*. juveniles). These analyses were performed with *adegenet* [45, 46] and *hierfstat* [47] packages with R [48]. We also estimated multilocus heterozygosity (*MLH*) with the *inbreedR* [49] package. To compare the data sets, we calculated the significant differences using a Wilcoxon test with the *ggpubr* package, and all the results were plotted using the *ggplot2* package in R.

### Inbreeding and kinship

To understand how individuals within and among populations were related, we estimated the inbreeding coefficient (*F*_*IS*_) with the *hierfstat* package. Additionally, we calculated for each sample the identity-by-descendent, specifically the *Fhat3* index, based on the correlation between uniting gametes, with PLINK v.1.9 software.

With VCFtools v.0.1.15 [42], we performed a relationship analysis using the *relatedness phi* index [50] which is based on the KING algorithm (Kinship-base Inference of Genome-wide). In this test, the *relatedness* value can be interpreted as the probability of finding identical alleles by randomly sampling one allele from each heterozygous individual. The *relatedness* index equal to 0 means that individuals are unrelated, and a range from 0.0442 to 0.0884 corresponds to a relationship in 3^rd^ degree, full siblings or 2^nd^ degree from 0.0885 to 0.1774, parent–offspring or 1^st^ degree from 0.1775–0.354 and a *relatedness* >0.350 corresponds to a monozygotic twin [50]. For easier visualization, all negative values were converted to 0.

### Genetic structure and recent gene flow

To assess significant differences between the cultivated and wild samples, we conducted a series of complementary analyses. The first test was a principal component analysis (PCA) calculated with *adegenet* [45, 46] package in R. Then we used *F*_*ST*_ pairwise index, to estimated confidence intervals and *p-values* according to the method proposed by Wright [51] and updated by Weir and Cockerham [52]; this analysis was done using R package *StAMPP* [53] with 1000 bootstraps and with two data set (by sampling site and by management). Additionally, we reconstructed the relationships among samples with a UPGMA tree using *poppr* R package. Also, we performed an isolation by distance test to assess the spatial component of the population structure using the *vegan* [54] R package. For the Mantel test, we used the genetic distance (*F*_*ST*_) among sampling sites and geographic distance (km) estimated using Geographic Distance Matrix Generator [55] (https://biodiversityinformatics.amnh.org/open_source/gdmg/).

To understand the admixture between cultivated, nursery and wild plants, we performed a sampled assignment analysis using ADMIXTURE v.1.23 [56, 57], with different numbers of clusters (*K* 1 to 10), with three replicates for each K value and 2000 bootstraps. The best *K* value was determined using the likelihood and cross-validation error. The ancestry values for each sample were plotted using the *ggplot2* R package.

To trace the genetic and geographic origin of cultivated and nursery plants, we used BayesAss version 3.0.4 (BA3-SNPs). This program allows the estimation of recent migration using next-generation sequence data [58]. We performed two independent runs, the first one according to the type of management (wild, cultivated, and nursery) and the second by locality (sampling site). To optimize the analyses, we adjusted the migration parameters at 0.6 and inbreeding at 0.2, as suggested by the software’s manual. The iteration number was set up at 50000000, with 1000000 additional iterations considered as *burn-in*.

### Outlier loci detection and annotation

We used two complementary approaches to detect outlier loci. The data set was divided into three groups corresponding to the management type (wild, cultivated, and nursery). First, we used BayeScan v2.0 [59, 60], which identifies candidate *loci* using differences in allele frequencies among populations. The parameters were set as follow: 10 interval thinning size, 5000 length of pilot runs, and 500 000 burn-in length. Then, using R software, we applied the Empirical Cumulative Distribution Function (*ecdf*), calculated the *p-value*, and adjusted it with *BH* = 0.05, or its alias false discovery rate (*fdr*), this adjustment controls the expected proportion of false discoveries [61].

The second analysis was performed using the R package *pcadapt*, which is based on a multi-dimensional approach that measures how distant each point is from the mean [62, 63]. This analysis performs principal component analysis and calculates *p-values* to test outliers based on the correlation between genetic variation and the first principal components [62, 63]. We estimated the first *K* principal components, testing different numbers of *K*, with the best fit in *K* = 3. Then we calculated the *p-values* and adjusted them with a *Bonferrroni* correction setting *alpha* at 0.001.

Subsequently, we used the annotated transcriptome of *A*. *tequilana* (GAHU00000000.1; [41]) by performing an alignment with Blast [64] using a dataset generated for the purpose of this study, which was made up of 16 annotations of species of the class Liliopsida: *Sorghum bicolor* (GCF_000003195.3), *Brachypodium distachyon* (GCF_000005505.3), *Setaria italica* (GCF_000263155.2), *Musa acuminata subsp*. *malaccensis* (GCF_000313855.2), *Elaeis guineensis* (GCF_000442705.1), *Oryza sativa* (GCF_001433935.1), *Ananas comosus* (GCF_001540865.1), *Asparagus officinalis* (GCF_001876935.1), *Triticum dicoccoides* (GCF_002162155.1), *Panicum hallii* (GCF_002211085.1), *Aegilops tauschii subsp*. *strangulata* (GCF_002575655.1), *Setaria viridis* (GCF_005286985.1), *Phoenix dactylifera* (GCF_009389715.1), *Dioscorea cayenensis subsp*. *rotundata* (GCF_009730915.1), *Zea mays* B73 (GCF_902167145.1) and the model species *Arabidopsis thaliana* (GCF_000001735.4); using an *e-value* of 0.00001.

The loci that were detected as outliers by BayeScan and *pcadapt* were mapped to the already annotated transcriptome so we could identify their function (biological, cellular, or molecular) and the associated protein through Gene Ontology (GO) and InterProScan [65].

## Results

### Sequencing and genotyping

We obtained 72 GB of raw data, with an average sequencing *phred* quality of 36.54% GC content, and a total of 5,759,792 raw reads. After initial quality filtering and SNP calling with Ipyrad software, 722,166 putative SNPs were derived. Subsequently, when we performed the quality filters using VCFtools, we obtained a total of 14,875 high-quality SNPs and 29,750 alleles, with 9.1% missing data.

Eighteen plants that presented low number of reads were excluded. The final data set consisted of 174 individuals, of which 104 were collected in Oaxaca state, Mexico, and 70 in Puebla state, Mexico. By management type, the final data set was 105 individuals from the wild category, 42 cultivated, and 27 from nurseries. Moreover, 147 plants were cataloged as adults and 27 as juveniles.

### Genetic diversity

The number of multilocus genotypes was *mlg* = 174 (Table 1), indicating that all organisms had a unique (different) genotype. The total heterozygosity for *A*. *potatorum* was *H*_*T*_ = 0.252. At the species level, the expected heterozygosity was greater than the observed heterozygosity (*H*_*E*_ = 0.232, *H*_*O*_ = 0.213). The expected heterozygosity at the sampling site level ranged between *H*_*E*_ = 0.194–0.248 (S1 Fig in S1 File). The highest heterozygosity was found in two wild populations, one from Oaxaca (Ox4-W: *H*_*E*_ = 0. 248) and another from Puebla (P1-W: *H*_*E*_ = 0.247). The cultivated population from Sola de Vega, Oaxaca (SV2-C) presented the lowest expected heterozygosity (*H*_*E*_ = 0.194). On the other hand, the observed heterozygosity ranged between *H*_*O*_ = 0.175–0.232. Populations with the highest observed heterozygosity were the nursery localities from San Juan Raya, Puebla (SJR1-N: *H*_*O*_ = 0.232), the two wild localities of the same site (SJR5-W: *H*_*O*_ = 0.231; SJR4-W: *H*_*O*_ = 0.230) and Tepalcatepec (Oax1-W: *H*_*O*_ = 0.227), while a wild population from Puebla (P4 -W, Valsequillo) presented the lowest observed heterozygosity (*H*_*O*_ = 0.175) (S1 Fig in S1 File).

**Table 1:** Genomic diversity index in *Agave potatorum* calculated with 14,875 SNPs. *mlg* = multilocus genotype; *PA* = private alleles; *H*_*O*_ = observed heterozygosity; *H*_*E*_ = expected heterozygosity; *MLH* = multilocus heterozygosity, *F*_*IS*_ and *Fhat3* = inbreeding coefficient, SD after ±.

*Diversity index*	*Management*			*Age*		*Full data set*
	*Wild*	*Cultivated*	*Nursery*	*Adults*	*Youth*	
***mlg***	105	42	27	NA	NA	174
***Private alleles***	11	0	0	NA	NA	11
***Ho***	0.213 ± 0.012	0.209 ± 0.005	0.227 ± 0.006	0.212 ± 0.011	0.227 ± 0.006	0.213
***HE***	0.234 ± 0.010	0.227 ± 0.013	0.244 ± 0.003	0.231 ± 0.011	0.244 ± 0.003	0.252
***MLH***	0.214 ± 0.026	0.209± 0.006	0.229 ± 0.016	0.212 ± 0.023	0.229 ± 0.016	0.215
*F*_*IS*_	0.029 ± 0.043	0.019 ± 0.062	0.053 ± 0.021	0.026 ± 0.049	0.053 ± 0.021	0.082
***Fhat3***	0.157 ± 0.009	0.150 ± 0.021	0.121 ± 0.058	0.155 ± 0.085	0.121 ± 0.058	0.15
***N***	105	42	27	147	27	174

**Fig 2:**
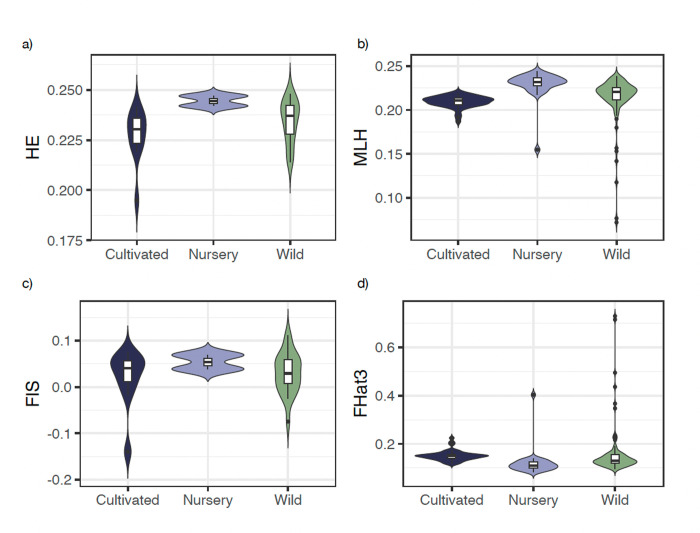
Genomic diversity index. Violin graph that shows the distribution of the genomic diversity; within each graph, there is a box plot showing the mean (intermediate line) and the variance of the data. a) expected heterozygosity (*H*_*E*_); b) Multilocus heterozygosity (*MLH*); c) Inbreeding index (*F*_*IS*_); d) *Fhat3*, per type of management: cultivated, nursery, and wild, estimated with 14 875 SNPs.

The expected heterozygosity was similar in the wild and nursery groups without significant differences (Wild: *H*_*E*_ = 0.234, SD = 0.010; Nursery *H*_*E*_ = 0.244 SD = 0.001). Cultivated samples presented significantly lower genetic diversity (*H*_*E*_ = 0.227, SD = 0.013) compared to nursery (*Wilcoxon test; P* = 0.0303), but without significant differences between cultivated and wild groups (*Wilcoxon test; P* = 0.163) (Fig 2A and S2 Table in S1 File).

The analysis by management category showed that the number of private alleles (*PA*) in wild localities was *PA* = 11, while the nursery and the cultivated groups had no private alleles (Table 1).

In the individual-based multilocus heterozygosity (*MLH*) analysis (S1 Fig in S1 File), the most diverse sampling sites were populations from the nursery locality of San Juan Raya, Puebla (SJR1-N: *MLH* = 0.244, SD = 0.008), followed by the wild localities SJR5-W (*MLH* = 0.237, SD = 0.002) and SJR4-W (*MLH* = 0.232, SD = 0.007). A wild population of Puebla (P4-W) had the lowest value of MLH (*MLH* = 0.115, SD = 0.058).

When we compared management types (Fig 2B and Table 1), the highest multilocus diversity was obtained for the nursery group (*MLH* = 0.229 SD = 0.016), followed by wild samples (*MLH* = 0.214; SD = 0.014) while the plants from cultivated sites presented the lowest multilocus heterozygosity (*MLH* = 0.209; SD = 0.006). We found significant differences among all comparisons based on management type; wild *vs*. cultivated (*Wilcoxon test; P* = 2.76E-07), wild *vs*. nursery (*Wilcoxon test; P =* 3.83E-07) and cultivated *vs*. nursery (*Wilcoxon test; P* = 1.14E-13) (S2 Table in S1 File).

### Inbreeding and kinship

The inbreeding index (*F*_*IS*_) showed considerable variation among samples (S1 Fig in S1 File), ranging from -0.139 to 0.110 (mean 0.028; SD = 0.048). One wild population from Oaxaca “Cuicatlán” (Cu4-W), and another from Puebla (P4-W) had the highest positive inbreeding coefficient (*F*_*IS*_ = 0.110 and *F*_*IS*_ = 0.100, respectively). In contrast, one cultivated population from Oaxaca: Sola de Vega (SV2-C), and wild localities from Cuicatlán (Cu7-W, Cu3-W), Ixtlán (Ix-W) and the cultivated samples from Matatlán (Mat-C), had an excess of heterozygotes.

When performing the analysis by management category (Fig 2A and Table 1), the highest *F*_*IS*_ values were obtained for the nursery samples (*F*_*IS*_ = 0.053, SD = 0.021), followed by the wild samples (*F*_*IS*_ = 0.029, SD = 0.043) and finally, the cultivated localities (*F*_*IS*_ = 0.019 SD = 0.062). We found significant differences (S2 Table in S1 File) between nursery and cultivated samples (*Wilcoxon test; P* = 0.0303) and nursery and wild samples (*Wilcoxon test; P* = 0.029).

All categories of *A*. *potatorum* presented considerable levels of inbreeding. We obtained an average inbreeding index of *Fhat3* = 0.150 (SD = 0.082). The highest coefficient of inbreeding was obtained for wild populations (*Fhat3* = 0.157, SD = 0.009), followed by cultivated populations (*Fhat3* = 0.150, SD = 0.021) and finally, nursery populations (*Fhat3* = 0.121, SD = 0.058), with significant differences between and among all types of management (Fig 2D and S2 Table in S1 File).

The kinship analysis (*relatedness phi*) showed that, in general, the sampled individuals were unrelated (S2 Fig in S1 File). When carrying out the analysis by management type, in wild localities we estimated an average *r* of 0.005, in the cultivated plants *r* = 0.013, and in the nursery plants *r =* 0.028. In the case of the nursery group, all the seeds were collected from different mother plants, so it was expected that they had low relatedness. These estimates correspond to the categories of third-degree relationships and unrelated.

At the sampling site level (S2 Fig in S1 File), a third-degree relationship was found in different localities: 1) between individuals from the nursery of San Juan Raya, Puebla (SJRN-1 and SJRN-2); 2) in the wild populations from Puebla (SJR4 and SJR5); 3) between the wild locality from Cuicatlán (Cu7); 4) in the cultivated samples from the locality from Sola de Vega (SV4-C) and 5) a mezcal factory (Palenque “Mc-C”) from Oaxaca Valley.

### Genetic structure and recent gene flow

**Fig 3:**
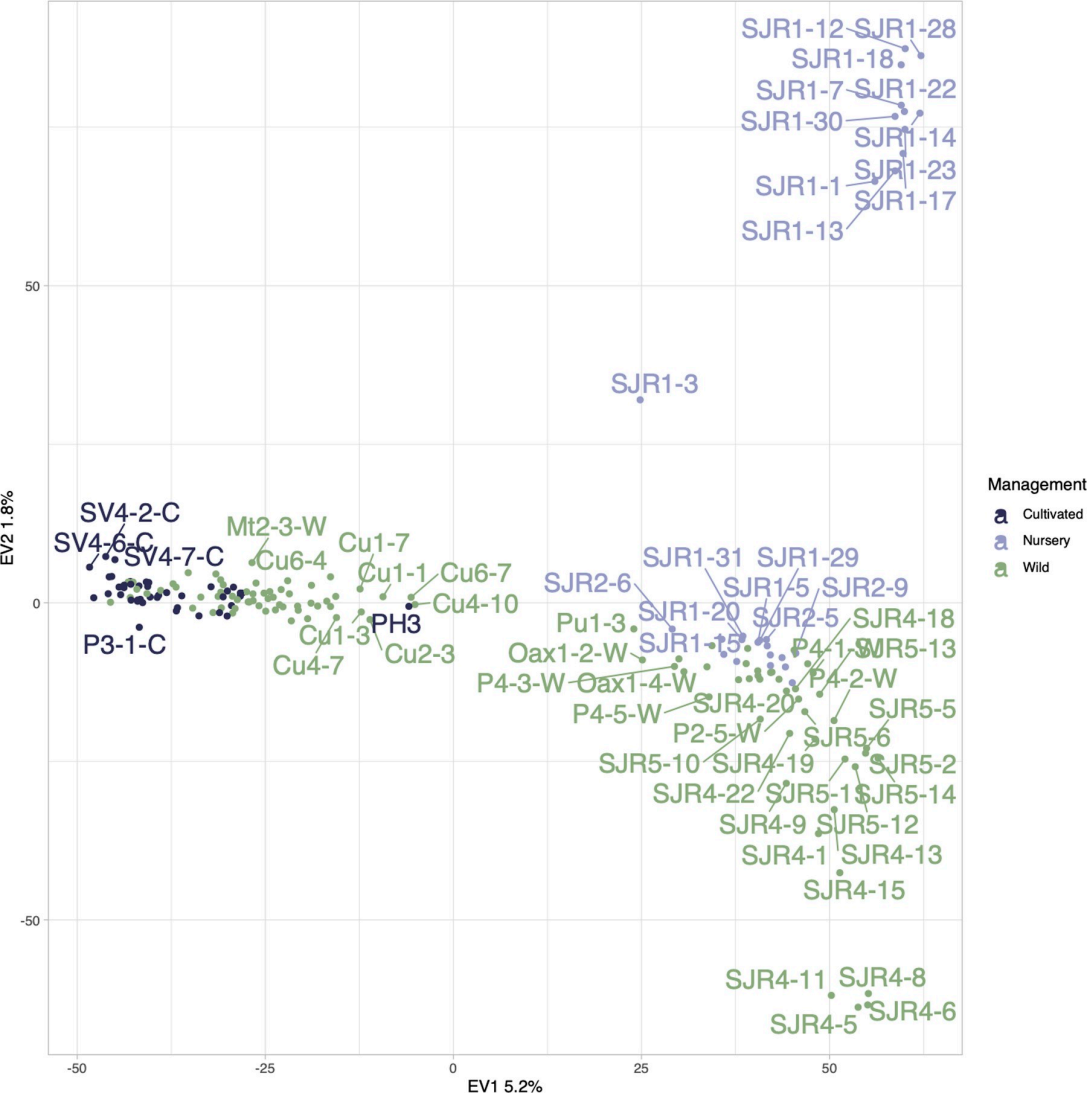
Principal component analysis (PCA) of *Agave potatorum* individuals, cultivated samples in dark purple, nursery in purple and wild in green estimated with 14 875 SNPs.

A principal component analysis (PCA) revealed three genetic clusters in our data set. Nevertheless, the variance explained by the first two axes was not high, with EV1 at 5.2% and EV2 at 1.8%. The first group, in the upper right quadrant of the plot (Fig 3), contained individuals from the nursery site from San Juan Raya, Puebla (SJR1-N). The second cluster, in the lower right, was formed by the nursery localities (SJR1-N and SJR2-N), some wild samples from the same geographic region (SJR4-W and SJR5-W), and several wild localities from Oaxaca and Puebla (Oax1-W, Pu1-W, P2-W, P4-W). The last cluster was composed of all the cultivated samples from Oaxaca and Puebla, and samples from the wild localities of Cuicatlán and Matatlán, Oaxaca.

The overall genetic differentiation among sample localities was relatively low, with *F*_*ST*_ = 0.0796. The pairwise values between localities ranged from *F*_*ST*_ = 0.005 to 0.186 (S3A Fig in S1 File). The sampling sites of San Juan Raya in Puebla (SJR) had very low differentiation (*F*_*ST*_ = 0.005 to 0.159) among them. The site exhibiting the most divergence was SV2-C, a cultivated sample from Sola de Vega, which had the greatest difference with respect to the other samples, particularly with the cultivated site of Puebla which was the only population found in a monoculture (P3-C) (*F*_*ST*_ = 0.159).

When we analyzed the genetic differentiation considering the different types of management, we found the highest genetic difference between cultivated and nursery, *F*_*ST*_ = 0.080 (*p-value =* 0). Wild *vs*. nursery *F*_*ST*_ = 0.031 (*p-value =* 0) was intermediate. The lowest differentiation was found between cultivated and wild samples *F*_*ST*_ = 0.023 (*p-value =* 0).

In the UPGMA analysis (S3B Fig in S1 File), we found three main groups. The most divergent groups contained the wild individuals from Puebla (P4-5W) and Cuicuatlán (Cu6-7, CU4-10, Cu1-3 Cu4-2), Oaxaca. The second group was the largest, comprising the cultivated localities of Puebla (P3-C) and cultivated (Palenque) localities of Oaxaca (Mc-C, PCon, PH, Car, SCM), the wild and cultivated populations of Sola de Vega (SV-C, SV-W), and the wild localities of Matatlán (Mt-W), Cuicatlán (Cu-W), Ixtlán (Ix-W) and Oaxaca (Oax-W). The last group was almost entirely formed by wild populations from Oaxaca, wild and nursery localities of San Juan Raya (SJR) and wild populations of Puebla (P-W). An analysis of isolation by distance showed a strong correlation (*r* = 0.48, *p* < 0.001), suggesting the importance of distance in structuring *A*. *potatorum* populations (S4 Fig in S1 File).

**Fig 4:**
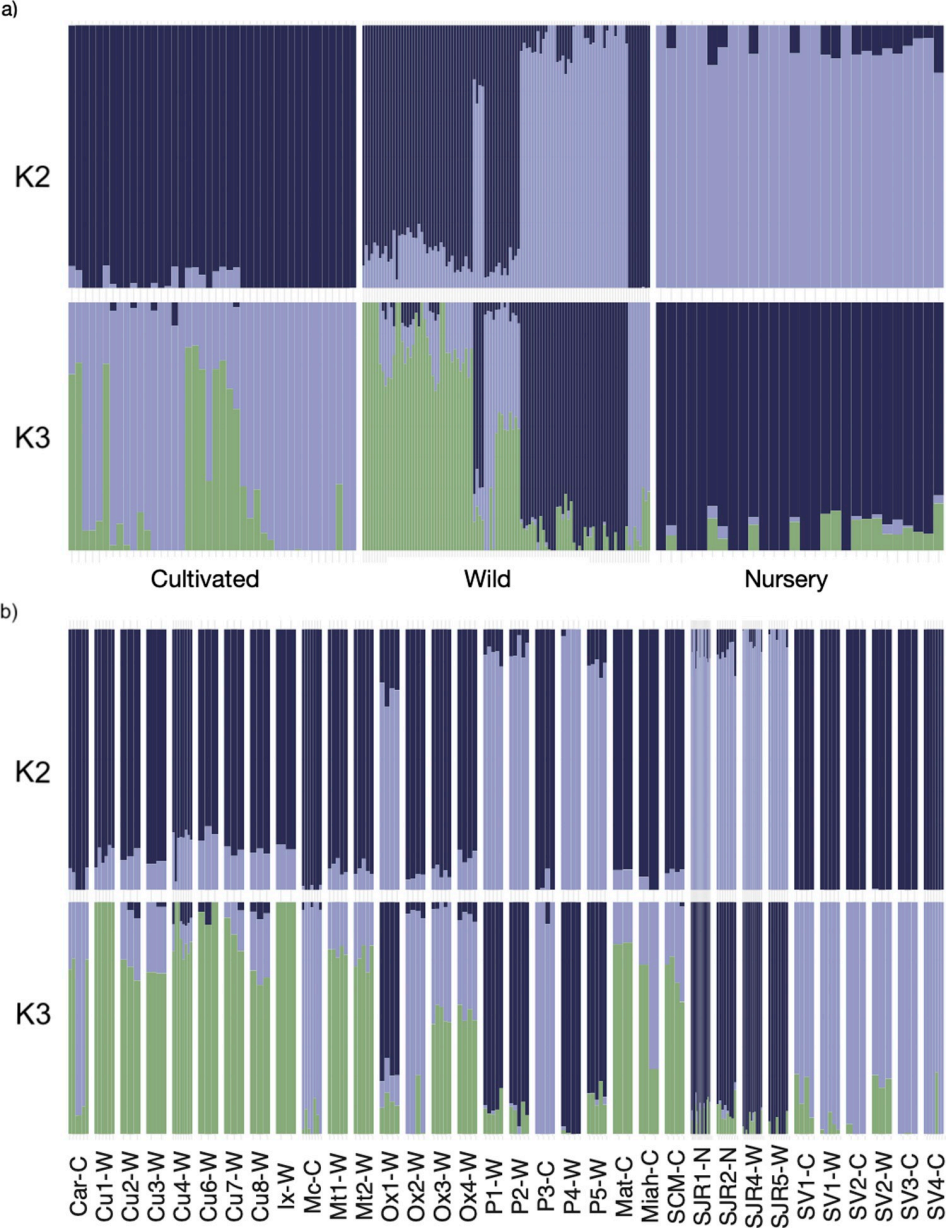
Admixture analysis showing the assignment probability of individuals from *Agave potatorum* (*K = 2* and *K = 3*) a) by type of management: (cultivated, nursery, and wild), and b) by locality.

Admixture analysis showed different levels of assignment (Fig 4A and 4B). *K* = 3 was the most probable *K* (CV = 0.49875), followed by *K* = 2 (CV = 0.49945) (S3 Table in S1 File). At *K = 2* (Fig 4A), the wild localities were divided into two genetic groups, each corresponding to a state (i.e., Oaxaca or Puebla). The nursery localities were in the same genetic pool (pale blue in *K* = 2, Fig 4A) as the wild samples from Puebla. In contrast, the cultivated individuals shared alleles with wild samples from Oaxaca (in dark blue in *K* = 2, Fig 4A). In *K = 3*, we identified a similar pattern to *K = 2*, only with more detail; the nursery and cultivated localities present their gene pool (pale blue for cultivated, dark blue for nursery, K = 3 Fig 4A), and each group with different derived alleles from the wild populations.

To understand better the ancestry, we made the plots of *K = 2* and *K = 3* by locality (Fig 4B). In *K = 2*, all the localities from Sola de Vega grouped in their own genetic pool (in dark blue in Fig 4B); likewise for the populations of San Juan Raya (SJR) and the wild localities of Puebla (P1-W, P2-W, P3-W, P4-W) (in pale blue in the Fig 4B). In contrast, in *K* = 2 the populations from wild localities of Oaxaca (Cuicatlán, Ixtlán, Matatlán, Ox2-W, Ox3-W, and Ox4-W) and the cultivated localities (Mc-C, Matatlán, Miahuatlán, and Santa Catarina Minas) had alleles shared between the two large gene pools.

In *K = 3* (Fig 4B) we observed that the wild localities from Cuicatlán presented their own genetic pool (in green in this figure), forming a group among themselves and with a geographically close locality from Ixtlán (Ix-W), with alleles shared with the wild (Ox1-W, Ox2-W, Ox3-W, Ox4-W) and cultivated (Mat-C, Miahu-C, SCM-C) populations of Oaxaca, while the localities of Sola de Vega (in pale blue in this figure) and San Juan Raya (in dark blue) had their own gene pools.

**Fig 5:**
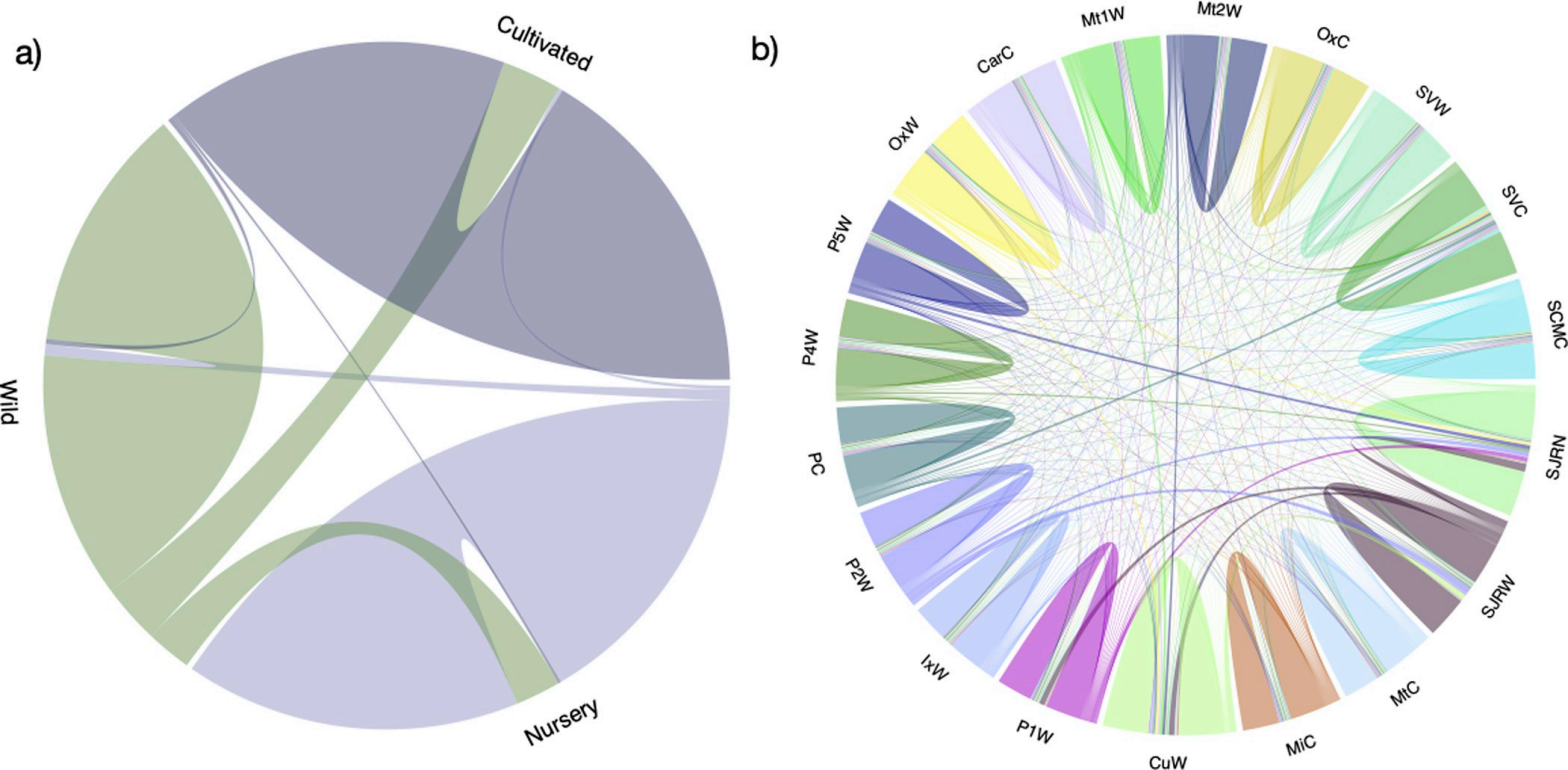
Migration rates estimated with BA3 software and 14,875 SNPs. a) migration rate per management type: cultivated, nursery, and wild; b) Migration rates per locality.

Gene flow analysis revealed an interesting pattern (Fig 5). In the site-level analysis (Fig 5B), we estimated a low proportion of migrants within each site, ranging from 0.007 to 0.098 (S4 Table in S1 File). For instance, the most considerable fraction of migrants (*m* = 0.099; SD = 0.042) was found from populations of Sola de Vega (SVC) to different locations, for example, the cultivated populations of San Dionisio Ocotlán (CarC: *m* = 0.065, SD = 0.027) and Oaxaca (OxC: *m* = 0.060, SD = 0.028).

Gene flow analysis at a management type level (wild, cultivated, and nursery), revealed a higher proportion of migration from wild populations (*m* = 0.171; SD = 0.016) towards cultivated localities, followed by migration from wild populations to nursery sites (*m* = 0.129; SD = 0.016) (Fig 5A and S5 Table in S1 File).

### Outlier loci detection and annotation

Our analysis of outliers only identified one significant SNP with BayeScan software, with *BH* = 0.05 and a *p-value* of 0.01. (S5 Fig in S1 File) Nevertheless, it was not possible to annotate it.

**Fig 6:**
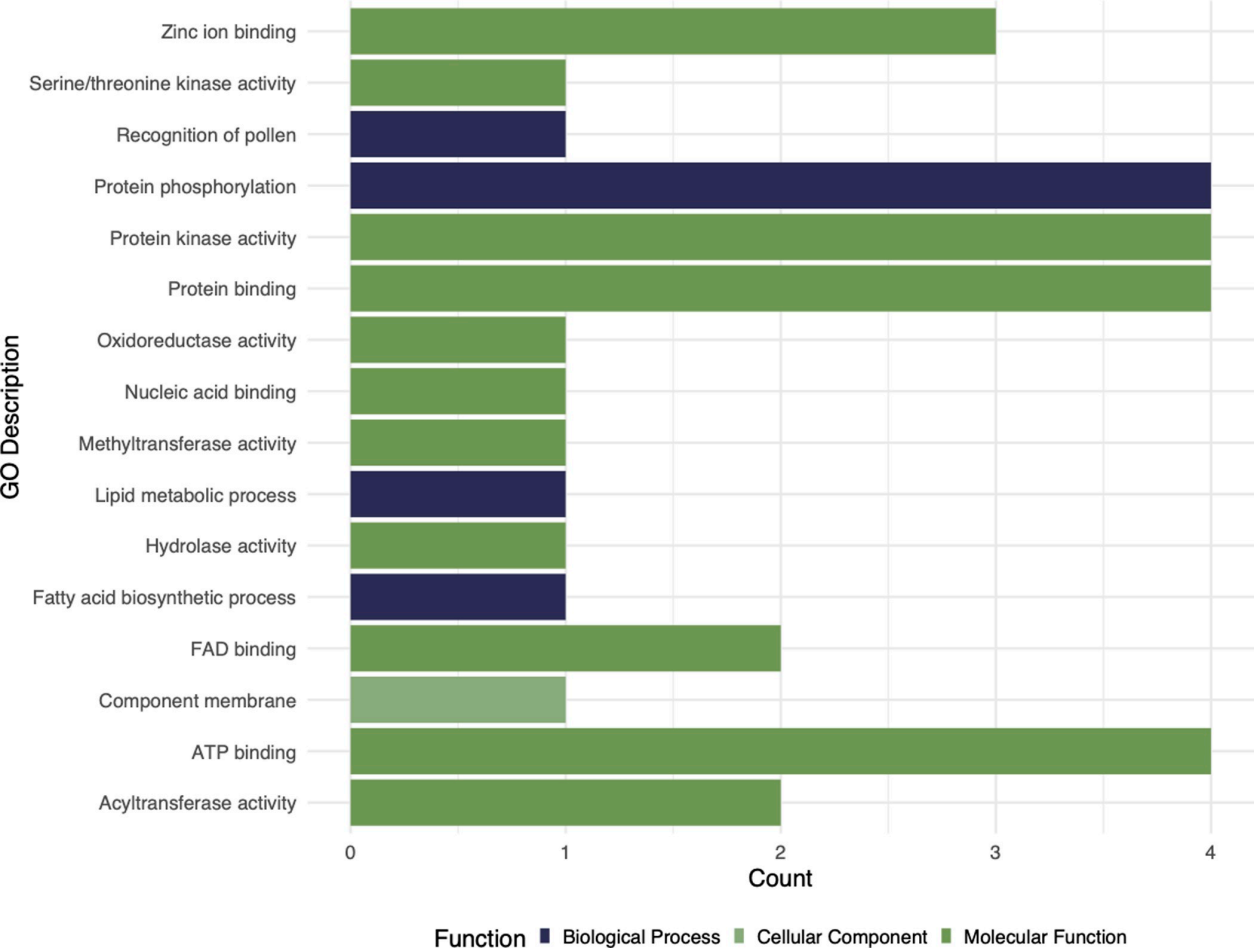
Annotation with Gene Ontology of outlier loci detected with pcadapt with possible selection signs in *Agave potatorum* from the Mexican states of Oaxaca and Puebla.

On the other hand, we identified 32 candidate outlier loci using *pcadapt* (Fig 6 and S6 Table in S1 File). The functional activity was annotated for the candidate loci, and 12 of these outliers belonged to the “No GO terms” (terms of Gene Ontology) category. Most annotated candidate loci associated to molecular functions (75%), followed by biological processes (21.875%), and the rest to cellular components (3.125%). We found outliers related to methyltransferase activity (GO:0008168), protein kinase activity (GO:0004672), protein (GO:0005515) adenosine triphosphate (ATP) (GO:0005524) binding, zinc ion binding (GO:0008270), protein phosphorylation (GO:0006468), flavin adenine dinucleotide (FAD) binding (GO:0006633), acyltransferase activity (GO:0016746), recognition of pollen (GO:0048544) and membrane components (GO:0016020).

## Discussion

### Genetic diversity

For plants with a long generation time and under incipient domestication, such as *Agave* species, understanding the evolution process and estimating their genetic diversity is essential for their conservation and management. Artificial selection creates bottlenecks in populations, and plants under cultivation generally represent only a limited portion of number of wild plants, which reduces the effective population size decreasing in turn their levels of genetic diversity. Therefore, it is crucial to set a baseline for diversity and differentiation metrics within these species.

In the case of *A*. *potatorum*, we found that the genetic diversity measured as expected heterozygosity was higher in the nursery and wild plants than in the cultivated samples (*H*_*E*_ = 0.244, *H*_*E*_ = 0.234, *H*_*E*_ = 0.227, respectively). We did not find private alleles in the cultivated or in the nursery plants, indicating that the plants under management came very recently from wild populations.

Expected heterozygosity values in *A*. *potatorum* (*H*_*T*_ = 0.252) were similar but lower than those previously reported for this species with dominant ISSR markers (i.e., *H*_*T*_ = 0.302 [16] and *H*_*T*_ = 0.294 [66]), and much lower than results based on microsatellites (SSRs), where very high genetic diversity was reported (wild: *H*_*T*_ = 0.87, populations under extraction, *H*_*T*_ = 0.72, and samples of a germplasm bank *H*_*T*_ = 0.69) [15, 67]. These differences may be due to differences in the mutation rates of the molecular markers used. Nevertheless, contrary to previous studies that did not detect a significant difference in genetic diversity between different management categories [15, 66, 67], we found that the cultivated samples of *A*. *potatorum* presented significant lower genetic diversity.

Similar diversity levels to the ones we obtained were reported in recent *Agave* studies using the same RADseq methodology. For instance, in *A*. *angustifolia* from Sonora, used to produce a type of mezcal called Bacanora, the value of genetic diversity (*H*_*T*_ = 0.250) was similar, with no differences between wild and cultivated plants [11]. In this species, similar management practices to Tobalá are used (i.e., incipient management and the introduction of plants and seeds from the wild). Our heterozygosity results were also similar to those reported using RADseq for the wild *A*. *angustifolia* from Oaxaca (*H*_*E*_ = 0.24), but lower than those founded in the cultivated *A*. *angustifolia* known as “Espadín” (*H*_*E*_ = 0.29) [68]. This last difference may result from different type of management practices between *A*. *angustifolia* “Espadín” and *A*. *potatorum*. *Agave angustifolia* has a long history of intensive clonal propagation as monoculture, with no genetic exchange with wild conspecifics. *Agave potatorum* management is recent and relies on wild plants and seeds. In contrast, we obtained a higher expected heterozygosity than the value estimated for the intensively cultivated, *A*. *tequilana* (*H*_*T*_ = 0.120) using RADseq [7].

*Agave potatorum* is a perennial species that takes 10 to 15 years to reach reproductive age. Due to this long generation time and seed harvest, the loss of diversity has apparently been gradual. Therefore, we argue that this species’ artificial selection and incipient management have not yet caused drastic differences between cultivated and wild individuals. In addition, *A*. *potatorum* has cross-pollination and usually reproduces only by seed and not clonally. Thus, we did not observe an excess of heterozygosity related to long-term clonal propagation found in other *Agave* species such as *A*. *tequilana* [7] and *A*. *angustifolia* [68].

In other perennial species, such as grapes [69], apples [70, 71], and cherries [27, 72] slight loss of diversity has been documented, suggesting that they maintain ~95% of the neutral variation found in wild populations [27], which seems to be the case for *A*. *potatorum*.

### Inbreeding and kinship

Inbreeding in cultivated crops may be common, it can result from either selection, self-pollination, or mating with closely related individuals within the same cultivated plot [73]. The genetic consequence of inbreeding is an increase in homozygosity [74] and, in consequence, the expression of deleterious recessive alleles known as inbreeding depression.

In previous studies, a high level of inbreeding was reported in *A*. *potatorum* by using microsatellites (*F*_*IS*_ = 0.267 [15]). In contrast, we estimated lower inbreeding at the species level (*F*_*IS*_ = 0.0827 and *Fhat3* = 0.150), but we obtained significant differences between different types of management with *Fhat3* index (wild = 0.157; cultivated = 0.150; nursery = 0.121). We found positive levels of inbreeding (i.e., *F*_*IS*_ and *Fhat3* index) in several *A*. *potatorum* populations. This inbreeding is probably related to habitat fragmentation and isolation among populations, causing mating among relatives to be more frequent.

During our fieldwork, we observed extensive deforested areas with few or no plants, and only a handful of individuals at the reproductive stage. These factors, including habitat fragmentation, isolation, and inbreeding, can lead to reduced effective population sizes (*N*_*e*_), resulting in a loss of genetic variation [75–77]. Additionally, these plants thrive in specific climates, such as pine-oak forests and xerophytic scrublands; which further isolate them and limit their ability to reproduce with geographically distant populations.

In addition to the genetic problems that isolation and possible fragmentation generate, demographic and ecological issues [78–80] lead to a disruption in biotic interactions [77] (for example, plant-pollinator, or plant-microbiome relationships, especially association with mycorrhizas). For instance, in *Agave* spp., a density-dependent effect has been documented with their main pollinators, that are nectarivorous bats. Pollinators are more efficient and abundant in populations with a higher density of flowering plants [22]. A similar effect has been observed in the interaction with mycorrhizae, with greater efficiency in obtaining nutrients, minerals, and protection against root pathogens if the plants have a healthy microbiome [81].

We found a negative inbreeding index, indicating an excess of heterozygotes in two cultivated populations (SV2-C y Mat-C), and five wild localities (Cu7-W, Cu3-W, Ix-W, SJR5-W, SV1-W). In previously published works on the *Agave* genus, negative *F*_*IS*_ values have been found, especially in cultivated individuals of intensively managed species, such as *A*. *tequilana F*_*IS*_ = -0.042 [7] and *A*. *angustifolia* “Espadín” *F*_*IS*_ = -0.07 [68]. These values have been related to artificial selection for hybrid vigor and an accumulation of somatic mutations in the genome. However, due to the type of reproduction in *A*. *potatorum* (usually by seed) and incipient cultivation, it is possible that these negative values of the Inbreeding Coefficient (*F*_*IS*_) are related to the fact that individuals with different genotypes or phenotypes tend to mate, resulting in excess of heterozygotes [74], or that selection eliminates the more inbreed or homozygote individuals.

In the kinship analysis, we obtained very low values, most of them corresponding to unrelated individuals, which we consider to be the result of sexual reproduction and probably long-distance gene flow within this species.

### Genetic structure and recent gene flow

The genetic structure reflects the balance between population cohesion and the process of divergence [82]. In general terms, the genetic structure is related to mating and migration since the probability that two individuals mate depends mainly on the geographical location of the potential mate and the dispersal capabilities of the species in question [74].

*Agave* species typically exhibit low genetic structure and differentiation [2, 3], a characteristic attributed to their long generation time, reproduction and pollination by highly mobile pollinators, in particular by bats. Our results confirmed this pattern as we found low genetic differentiation among populations of *A*. *potatorum* (*F*_*ST*_ = 0.0796). Similar values have been reported in the same species in two studies using ISSRs (*F*_*ST*_ = 0.099 [16], *F*_*ST*_ = 0.079 [66]). These authors attributed this low genetic structure to the exchange of pollen driven by the nectar-feeding bat *L*. *yerbabuenae*. Nevertheless, higher genetic differentiation values were reported for the species based on the microsatellites study (SSRs: *F*_*ST*_ = 0.389); these results were attributed to ecological, geological, and climatic effects of the sampling area [15, 67].

In our study, the paired lowest values of genetic differentiation (*F*_*ST*_) were found among San Juan Raya (SJR) populations in the south of the state of Puebla, and Cuicatlán, Oaxaca; both localities belonging to the Tehuacán-Cuicatlán Biosphere Reserve. This is a protected natural area located in the southeast of Mexico characterized by its great floristic diversity and high number of endemic species [83]. These localities are geographically close, and their distribution seems continuous. In contrast, we observed habitat fragmentation and isolation among populations in the Central Valley of Oaxaca. These patterns were also observed in the UPGMA cluster tree, where all the samples from San Juan Raya form a unique cluster. Furthermore, isolation by distance analysis was high and significant (*r* = 0.48, *p* <0.001), indicating that geographically close populations in general tend to be more genetically similar. We suggest that the low genetic differentiation is indeed related to pollinators’ great mobility, mainly nectar-feeding bats. Indeed, we found genetic structure associated with geographic patterns, using the Admixture software, where the wild populations presented two gene pools (divided by Oaxaca and Puebla), with derived alleles towards nursery and cultivated localities.

At the finer scale, we found that genetic structure seems to be related to geography and ecology. For example, samples from Sola de Vega and Cuicatlán were found at a higher elevation (700–3,000 and 400–2,600 ms. n.m, respectively) along pine-oak forests, opposite to San Juan Raya, Puebla (average 1,700 ms. n.m), where the plants were collected in a xeric scrub. On the other hand, populations of the Central Valley, Oaxaca (700 a 1,600 ms.n.m.) were found at lower altitudes with a greater degree of deforestation and, therefore, less continuity and lower connectivity among populations.

Gene flow among populations limits genetic structure [82], helps to maintain genetic diversity and prevents inbreeding, which is especially important for small and fragmented populations [84]. Nevertheless, it requires that migrating individuals successfully contribute alleles to the mating pool of the population [74]. We estimated a higher gene flow from wild populations to cultivated sites, indicating that the plants under cultivation have been extracted from wild populations, or that the seed that originated them came from wild plants. However, at the population level, we found that gene flow is generally low. The highest inferred migration rate was in the localities of Sola de Vega and San Juan Raya; these populations are the best conserved and have less habitat fragmentation. As mentioned above, the main pollinators of *A*. *potatorum* are nectarivorous bats, including not only *Leptonycteris yerbabuenae*, also *Leptonycteris nivalis* (Saussure, 1860), and *Choeronycteris mexicana* (Tschudi, 1844) [22, 85]. The long-distance dispersal can counteract the effects of genetic differentiation, further providing a possible mechanism for introducing new alleles [86]. In these localities with less fragmentation, bats are usually more common, due to the dependent density effect.

### Outlier loci detection and annotation

We consider it is relevant to generate information about the adaptive processes and identify potentially valuable genes for the cultivation and management of *A*. *potatorum* and other *Agave* species. This research can aid in future management, genetic conservation, and possible improvement in the face of global change. This data would be particularly relevant since a decrease in genetic diversity and inbreeding related to domestication may eventually cause reductions in adaptive potential and plasticity [77]. Additionally, local genetic adaptation is crucial to maintain phenotypic diversity in wild populations [87].

We found that the highest number of outlier loci with possible selection signals were related to molecular processes, of which we think the most noteworthy was related to protein phosphorylation, which plays an essential role in signal transduction and has a vital role in the life cycle of plants [88–91]. We also detected outlier loci related to protein kinase activity. This is the most important protein in signaling pathways, and can transfer phosphate groups with a significant role in enzyme regulation, gene expression, and transduction [92]. It is important to highlight that we found a protein family membership with S-receptor-like serine/threonine-protein kinase (SRK) (IPR024171). This protein has an important function in plants, as described in *Brassica* [93–95], where it is involved in the self-incompatibility mechanism that prevents inbreeding in and thereby increases genetic diversity [95–97].

Additionally, we also found an outlier locus related to the recognition of pollen, which is also relevant to Self-incompatibility protein (SI) and has the inability to produce zygotes after self-pollination in a hermaphrodite plant [97]. It is important to remember that *Agave* species have protandrous hermaphrodite flowers, female and male sex are found in the same plant. However, the female gonads mature first than the male ones, limiting self-pollination [22].

Given the significance of organoleptic properties in *A*. *potatorum* during distillation, our focus was to identifying outliers related to these traits or those associated with sugar production. However, we did not detect any outliers related to these functions. Instead, we found outlier loci with potential signs of selection linked to genes involved in reproduction and response to abiotic stress that may be relevant to the local adaptation of both wild and cultivated plants.

The outlier analyses should be taken with caution, as they are based on genetic differences among populations. We suggest that in the future it would be beneficial to establish a database with gene annotations and functions for *Agave*, similar to databases available for other species like rice (http://rapdb.dna.affrc.go.jp/; http://rice.plantbiology.msu.edu/). This will require complete, well-resolved at chromosome levels genomes, transcriptomes, proteomes, and annotation of the proteins, at least in some *Agave* species, which for the moment have not yet been published.

### Conservation implications and breeding strategies

The uncontrolled extraction of wild individuals and seeds increases the extinction risk of the populations [19] and, eventually, of the complete species, so we suggest some urgent steps to minimize these possibilities. First of all, it will be important to reduce future habitat fragmentation and the loss of wild populations of the species. It is also essential to increase the number of nurseries and shade houses for *A*. *potatorum*. This has been done in San Juan Raya (SJR), Puebla, which is the case of a successful collaborative effort between researchers and peasant communities initiated eight years ago to mitigate the overexploitation of wild *Agave* plants. As a tangible result, ca. 4000 plants were transplanted to natural vegetation after two years of growing in a shade house. Plants were obtained from seeds from a local *A*. *potatorum* population that were collected from spatially distant individuals, separated for more than 100 meters. After four years, 75% of transplanted plants survived [Valiente-Banuet et al., unpublished data]. Although the agroecological management at the SJR nursery can be considered a success, other *A*. *potatorum* producers should be aware of the importance of reducing the relatedness among cultivated individuals. Seeds should be collected from different mother plants and localities. These practices will decrease inbreeding and maintain the genetic diversity of the cultivated individuals. Nevertheless, it is also essential to maintain local adaptation and avoid outcrossing depression; therefore, the ecological characteristics of the habitat should also be considered. We believe that a global breeding program can also be implemented for *A*. *potatorum* where the wild samples’ origin, initial genetic diversity and differentiation are registered. These practices will reduce inbreeding, inbreeding depression and serve as a gene bank for future breeding necessities and for adapting to the global changes.

On the other hand, successful *in situ* conservation efforts hinge on a careful consideration of the demography patterns of the wild populations, which can effectively serve as germplasm banks [26]. Additionally, the connectivity of these populations and the reproductive success of the plants relies mainly on the nectar feeding bats, making it essential to protect these primary pollinators.

Given the absence of significant genetic differences between wild and cultivated populations, we propose the reintroduction of a percentage of nursery-raised plants into the wild populations (as it has been done in SJR and SLA). Also, where appropriate, implementing agroecological management for this species *in situ*, [26] is recommend. This should take into account the consideration for biotic interactions, such as promoting establishment and survival with nurse plants [98], as well as facilitating seedling establishment [85].

## Conclusions

We found higher genetic diversity in the wild and nursery than in cultivated samples. Moreover, no private alleles related to management type (under cultivation and nursery) were found, meaning that most of the managed samples were recently derived from natural populations. Inbreeding levels varied widely among studied samples, apparently associated with isolation and fragmentation, but unrelated to human selection and management. Genetic differentiation at the species level was low. However, we found three genetic groups. Also, we identified higher gene flow in populations growing in less fragmented habitats. Furthermore, elevated gene flow from wild populations to the other two types of management was detected. Finally, we found loci with possible selection signals mainly related to biological processes and apparently to self-incompatibility.

As a conservation proposal, we suggest following the examples of SJR and SLA, with the production of plants in nurseries to avoid over-exploitation of wild plants and with the use of agroecological management, together with analysis of breeding and genetics, in order to trace the lineage of the plants and prevent inbreeding processes in populations of *A*. *potatorum*.

## Supporting information

S1 File Supporting information file containing multiple supporting figures (S1-S5 Fig) and tables (S1-S6 Tables). (DOCX)
