# Supplementary material for: Differences in the genomic diversity, structure, and inbreeding patterns in wild and managed populations of Agave potatorum Zucc. used in the production of Tobalá mezcal in Southern Mexico
Source: PLoS One. 2023 Nov 16;18(11):e0294534. doi: 10.1371/journal.pone.0294534 (PMC10653438; doi:10.1371/journal.pone.0294534)
Supplement: S1 File — (DOCX) [file pone.0294534.s001.docx]

| ***ID*** | ***N*** | ***Latitude*** | ***Longitude*** | ***State*** | ***Age*** | ***Management*** | ***Location*** |
| --- | --- | --- | --- | --- | --- | --- | --- |
| **Car-C** | 6 | 16.749128 | -96.668237 | Oaxaca | Adult | Cultivated | Palenque in San Dionisio Ocotlán |
| **Cu1-W** | 6 | 17.57341667 | -96.95055556 | Oaxaca | Adult | Wild | Cuicatlán |
| **Cu2-W** | 3 | 17.50824354 | -96.93751034 | Oaxaca | Adult | Wild | Cuicatlán |
| **Cu3-W** | 2 | 17.55427074 | -96.94435076 | Oaxaca | Adult | Wild | Cuicatlán |
| **Cu4-W** | 8 | 17.21525 | -96.75911111 | Oaxaca | Adult | Wild | Cuicatlán |
| **Cu6-W** | 3 | 17.6221666 | -96.92044445 | Oaxaca | Adult | Wild | Cuicatlán |
| **Cu7-W** | 2 | 17.46008333 | -96.93794444 | Oaxaca | Adult | Wild | Cuicatlán |
| **Cu8-W** | 4 | 17.61064595 | -96.9266945 | Oaxaca | Adult | Wild | Cuicatlán |
| **Ix-W** | 2 | 17.3113923 | -96.53825536 | Oaxaca | Adult | Wild | Ixtlán de Juarez |
| **Mat-C** | 3 | 16.8116367 | -96.3017352 | Oaxaca | Adult | Cultivated | Palenque in Matatlán |
| **Mc-C** | 7 | 17.139285 | -96.742115 | Oaxaca | Adult | Cultivated | Palenque in Oaxaca Valley |
| **Miah-C** | 2 | 16.41599098 | -96.53322274 | Oaxaca | Adult | Cultivated | Palenque in Miahuatlán |
| **Mt1-W** | 5 | 16.826132 | -96.367606 | Oaxaca | Adult | Wild | Matatlán |
| **Mt2-W** | 5 | 16.756968 | -96.342714 | Oaxaca | Adult | Wild | Matatlán |
| **Ox1-W** | 4 | 18.089637 | -97.689198 | Oaxaca | Adult | Wild | Tepalcatepec |
| **Ox2-W** | 4 | 17.57124 | -97.402094 | Oaxaca | Adult | Wild | San Juan Teposcolula |
| **Ox3-W** | 5 | 17.268943 | -96.986144 | Oaxaca | Adult | Wild | The Carbonera |
| **Ox4-W** | 4 | 17.292123 | -96.99909 | Oaxaca | Adult | Wild | El correo |
| **P1-W** | 5 | 18.978837 | -98.1875969 | Puebla | Adult | Wild | San Jose El Rincon |
| **P2-W** | 5 | 18.731526 | -97.530966 | Puebla | Adult | Wild | San Martin Esperilla |
| **P3-C** | 4 | 18.889153 | -98.112564 | Puebla | Adult | Cultivated | Tepenene (Monoculture) |
| **P4-W** | 4 | 18.948437 | -98.132352 | Puebla | Adult | Wild | Valsequillo |
| **P5-W** | 5 | 18.236143 | -97.553598 | Puebla | Adult | Wild | Acatepec |
| **SCM-C** | 4 | 16.750618 | -96.61702 | Oaxaca | Adult | Cultivated | Palenque in Santa Catarina Minas |
| **SJR1-N** | 19 | 18.30413796 | -97.63566904 | Puebla | Youth | Nursery | SJR agorecological plantation |
| **SJR2-N** | 8 | 18.31464199 | -97.61708996 | Puebla | Youth | Nursery | SJR Greenhouse |
| **SJR4-W** | 12 | 18.28826599 | -97.60372301 | Puebla | Adult | Wild | El Cualache |
| **SJR5-W** | 8 | 18.30457499 | -97.63549302 | Puebla | Adult | Wild | Near agorecological plantation |
| **SV1-C** | 4 | 16.45595 | -96.952453 | Oaxaca | Adult | Cultivated | Sola de Vega |
| **SV1-W** | 5 | 16.573544 | -96.933068 | Oaxaca | Adult | Wild | Sola de Vega |
| **SV2-C** | 3 | 16.464633 | -96.949452 | Oaxaca | Adult | Cultivated | Sola de Vega |
| **SV2-W** | 3 | 16.579074 | -96.908083 | Oaxaca | Adult | Wild | Sola de Vega |
| **SV3-C** | 3 | 16.464633 | -96.949452 | Oaxaca | Adult | Cultivated | Sola de Vega |
| **SV4-C** | 7 | 16.481723 | -96.95909 | Oaxaca | Adult | Cultivated | Sola de Vega |

**S1 Table.** Metadata of the collected localities of *Agave potatorum.*

**S2 Table.** Wilcoxon test for different types of management in *Agave potatoum* calculated with 14,875 SNPs.

| ***Wilcoxon test*** | | | | |
| --- | --- | --- | --- | --- |
| ***Group 1*** | ***Group 2*** | ***p*** | ***p.adj*** | ***p.signif*** |
| ***MLH vs Management*** | | | | |
| **Cultivated** | Wild | 2.76E-07 | 3.80E-07 | **** |
| **Cultivated** | Nursery | 1.14E-13 | 3.40E-13 | **** |
| **Wild** | Nursery | 3.83E-07 | 3.80E-07 | **** |
| ***MLH vs Age*** | | | | |
| **Adult** | Youth | 3.5E-09 | 3.5E-09 | **** |
|  |  |  |  |  |
| ***Fhat3 vs Management*** | | | | |
| **Cultivated** | Wild | 0.00109 | 1.10E-03 | **** |
| **Cultivated** | Nursery | 7.52E-09 | 2.30E-08 | **** |
| **Wild** | Nursery | 0.000013 | 1.90E-05 | **** |
| ***Fhat3 vs Age*** | | | | |
| **Adult** | Youth | 0.000000236 | 0.00000024 | **** |
|  |  |  |  |  |
| ***FIS vs Management*** | | | | |
| **Cultivated** | Wild | 0.984 | 0.98 | ns |
| **Cultivated** | Nursery | 0.485 | 0.73 | ns |
| **Wild** | Nursery | 0.406 | 0.73 | ns |
| ***FIS vs Age*** | | | | |
| Adult | Youth | 0.392 | 0.39 | ns |
|  |  |  |  |  |
| ***Ho vs Management*** | | | | |
| **Cultivated** | Wild | 0.176 | 0.18 | ns |
| **Cultivated** | Nursery | 0.0303 | 0.091 | * |
| **Wild** | Nursery | 0.087 | 0.13 | ns |
| ***Ho vs Age*** | | | | |
| **Adult** | Youth | 0.0428 | 0.043 | * |
|  |  |  |  |  |
| ***He vs Management*** | | | | |
| **Cultivated** | Wild | 0.163 | 0.18 | ns |
| **Cultivated** | Nursery | 0.0303 | 0.091 | * |
| **Wild** | Nursery | 0.181 | 0.18 | ns |
| ***He vs Age*** | | | | |
| **Adult** | Youth | 0.0891 | 0.089 | ns |

**S3 Table.** Cross-validation error in K values, using ADMIXTURE v.1.23 in *Agave potatorum* calculated with 14,875 SNPs.

| ***K*** | ***CV error*** |
| --- | --- |
| K=1 | 0.52542 |
| K=2 | 0.49945 |
| K=3 | 0.49875 |
| K=4 | 0.50314 |
| K=5 | 0.50132 |
| K=6 | 0.50886 |
| K=7 | 0.51828 |
| K=8 | 0.52925 |
| K=9 | 0.53634 |
| K=10 | 0.55666 |

**S4 Table.** Migration rate by management (wild, cultivated, and nursery) in *Agave potatorum* calculated with 14,875 SNPs, using BayesAss version 3.0.4.

| ***Pop1*** | ***Pop2*** | ***Migration rate*** | ***SD*** |
| --- | --- | --- | --- |
| Nursery | Nursery | 0.9571 | 0.0197 |
| Wild | Nursery | 0.129 | 0.0156 |
| Cultivated | Nursery | 0.0075 | 0.0073 |
| Nursery | Wild | 0.0322 | 0.0174 |
| Wild | Wild | 0.7001 | 0.0101 |
| Cultivated | Wild | 0.0146 | 0.01 |
| Nursery | Cultivated | 0.0108 | 0.0103 |
| Wild | Cultivated | 0.1709 | 0.016 |
| Cultivated | Cultivated | 0.9779 | 0.0121 |

**S5 Table.** Migration rate by locality in *Agave potatorum* calculated with 14,875 SNPs, using BayesAss version 3.0.4.

| ***Pop1*** | ***Pop2*** | ***Migration rate*** | ***SD*** |
| --- | --- | --- | --- |
| SJRN | SJRN | 0.8651 | 0.0234 |
| SJRW | SJRN | 0.1525 | 0.0237 |
| MtC | SJRN | 0.0159 | 0.0152 |
| MiC | SJRN | 0.0159 | 0.0152 |
| CuW | SJRN | 0.0071 | 0.0068 |
| P1W | SJRN | 0.0827 | 0.0291 |
| IxW | SJRN | 0.0158 | 0.0151 |
| P2W | SJRN | 0.0802 | 0.0303 |
| PC | SJRN | 0.0145 | 0.0141 |
| P4W | SJRN | 0.0433 | 0.0229 |
| P5W | SJRN | 0.0768 | 0.0314 |
| OxW | SJRN | 0.0465 | 0.0191 |
| CarC | SJRN | 0.0134 | 0.0129 |
| Mt1W | SJRN | 0.014 | 0.0134 |
| Mt2W | SJRN | 0.0137 | 0.0132 |
| OxC | SJRN | 0.0129 | 0.0125 |
| SVW | SJRN | 0.0124 | 0.012 |
| SVC | SJRN | 0.009 | 0.0087 |
| SCMC | SJRN | 0.0151 | 0.0144 |
| SJRN | SJRW | 0.007 | 0.0069 |
| SJRW | SJRW | 0.8019 | 0.0293 |
| MtC | SJRW | 0.0158 | 0.0151 |
| MiC | SJRW | 0.016 | 0.0151 |
| CuW | SJRW | 0.0072 | 0.0068 |
| P1W | SJRW | 0.0138 | 0.0133 |
| IxW | SJRW | 0.0159 | 0.0153 |
| P2W | SJRW | 0.1141 | 0.0135 |
| PC | SJRW | 0.0145 | 0.0138 |
| P4W | SJRW | 0.0146 | 0.0139 |
| P5W | SJRW | 0.0139 | 0.0131 |
| OxW | SJRW | 0.0092 | 0.009 |
| CarC | SJRW | 0.0133 | 0.0127 |
| Mt1W | SJRW | 0.0138 | 0.0132 |
| Mt2W | SJRW | 0.0139 | 0.0134 |
| OxC | SJRW | 0.0127 | 0.0124 |
| SVW | SJRW | 0.0124 | 0.012 |
| SVC | SJRW | 0.0091 | 0.009 |
| SCMC | SJRW | 0.0151 | 0.0144 |
| SJRN | MtC | 0.0073 | 0.0071 |
| SJRW | MtC | 0.0086 | 0.0086 |
| MtC | MtC | 0.683 | 0.0157 |
| MiC | MtC | 0.0158 | 0.015 |
| CuW | MtC | 0.0072 | 0.007 |
| P1W | MtC | 0.0139 | 0.0133 |
| IxW | MtC | 0.0157 | 0.015 |
| P2W | MtC | 0.0139 | 0.0133 |
| PC | MtC | 0.0145 | 0.0139 |
| P4W | MtC | 0.0143 | 0.0136 |
| P5W | MtC | 0.0139 | 0.0133 |
| OxW | MtC | 0.0092 | 0.0089 |
| CarC | MtC | 0.0134 | 0.0128 |
| Mt1W | MtC | 0.0139 | 0.0134 |
| Mt2W | MtC | 0.0139 | 0.0133 |
| OxC | MtC | 0.0127 | 0.0121 |
| SVW | MtC | 0.0124 | 0.0119 |
| SVC | MtC | 0.0089 | 0.0085 |
| SCMC | MtC | 0.0151 | 0.0144 |
| SJRN | MiC | 0.0069 | 0.0068 |
| SJRW | MiC | 0.0086 | 0.0084 |
| MtC | MiC | 0.016 | 0.0152 |
| MiC | MiC | 0.6983 | 0.0208 |
| CuW | MiC | 0.0071 | 0.0068 |
| P1W | MiC | 0.0138 | 0.0133 |
| IxW | MiC | 0.0157 | 0.0151 |
| P2W | MiC | 0.0138 | 0.0132 |
| PC | MiC | 0.0146 | 0.0139 |
| P4W | MiC | 0.0146 | 0.014 |
| P5W | MiC | 0.0139 | 0.0134 |
| OxW | MiC | 0.0092 | 0.0088 |
| CarC | MiC | 0.0135 | 0.0129 |
| Mt1W | MiC | 0.0139 | 0.0132 |
| Mt2W | MiC | 0.0139 | 0.0133 |
| OxC | MiC | 0.0129 | 0.0124 |
| SVW | MiC | 0.0123 | 0.0118 |
| SVC | MiC | 0.0091 | 0.009 |
| SCMC | MiC | 0.0152 | 0.0145 |
| SJRN | CuW | 0.0143 | 0.0098 |
| SJRW | CuW | 0.0084 | 0.0083 |
| MtC | CuW | 0.0454 | 0.0257 |
| MiC | CuW | 0.0317 | 0.0209 |
| CuW | CuW | 0.8719 | 0.0233 |
| P1W | CuW | 0.014 | 0.0134 |
| IxW | CuW | 0.0471 | 0.0255 |
| P2W | CuW | 0.0139 | 0.0133 |
| PC | CuW | 0.0146 | 0.0139 |
| P4W | CuW | 0.0144 | 0.0138 |
| P5W | CuW | 0.014 | 0.0134 |
| OxW | CuW | 0.0491 | 0.0296 |
| CarC | CuW | 0.0381 | 0.0217 |
| Mt1W | CuW | 0.0522 | 0.0262 |
| Mt2W | CuW | 0.0517 | 0.0264 |
| OxC | CuW | 0.0128 | 0.0124 |
| SVW | CuW | 0.0124 | 0.0119 |
| SVC | CuW | 0.0169 | 0.0123 |
| SCMC | CuW | 0.0152 | 0.0146 |
| SJRN | P1W | 0.007 | 0.007 |
| SJRW | P1W | 0.0085 | 0.0083 |
| MtC | P1W | 0.0158 | 0.015 |
| MiC | P1W | 0.0159 | 0.0151 |
| CuW | P1W | 0.0071 | 0.0071 |
| P1W | P1W | 0.6815 | 0.0143 |
| IxW | P1W | 0.0159 | 0.0151 |
| P2W | P1W | 0.014 | 0.0135 |
| PC | P1W | 0.0145 | 0.014 |
| P4W | P1W | 0.0146 | 0.0141 |
| P5W | P1W | 0.014 | 0.0133 |
| OxW | P1W | 0.0092 | 0.009 |
| CarC | P1W | 0.0135 | 0.013 |
| Mt1W | P1W | 0.0139 | 0.0133 |
| Mt2W | P1W | 0.014 | 0.0134 |
| OxC | P1W | 0.0129 | 0.0123 |
| SVW | P1W | 0.0124 | 0.012 |
| SVC | P1W | 0.009 | 0.0089 |
| SCMC | P1W | 0.0152 | 0.0146 |
| SJRN | IxW | 0.007 | 0.007 |
| SJRW | IxW | 0.0086 | 0.0083 |
| MtC | IxW | 0.0159 | 0.0151 |
| MiC | IxW | 0.0159 | 0.0152 |
| CuW | IxW | 0.0074 | 0.0071 |
| P1W | IxW | 0.0138 | 0.0133 |
| IxW | IxW | 0.6829 | 0.0156 |
| P2W | IxW | 0.014 | 0.0134 |
| PC | IxW | 0.0144 | 0.0139 |
| P4W | IxW | 0.0145 | 0.0139 |
| P5W | IxW | 0.0139 | 0.0134 |
| OxW | IxW | 0.0091 | 0.0089 |
| CarC | IxW | 0.0134 | 0.0128 |
| Mt1W | IxW | 0.0139 | 0.0135 |
| Mt2W | IxW | 0.0139 | 0.0133 |
| OxC | IxW | 0.0129 | 0.0123 |
| SVW | IxW | 0.0123 | 0.0118 |
| SVC | IxW | 0.0089 | 0.0088 |
| SCMC | IxW | 0.0151 | 0.0144 |
| SJRN | P2W | 0.007 | 0.0069 |
| SJRW | P2W | 0.0086 | 0.0084 |
| MtC | P2W | 0.0158 | 0.0152 |
| MiC | P2W | 0.0158 | 0.0151 |
| CuW | P2W | 0.007 | 0.0068 |
| P1W | P2W | 0.014 | 0.0135 |
| IxW | P2W | 0.0158 | 0.0151 |
| P2W | P2W | 0.6828 | 0.0178 |
| PC | P2W | 0.0145 | 0.0138 |
| P4W | P2W | 0.0145 | 0.0139 |
| P5W | P2W | 0.0138 | 0.0132 |
| OxW | P2W | 0.0092 | 0.0091 |
| CarC | P2W | 0.0134 | 0.0128 |
| Mt1W | P2W | 0.0139 | 0.0132 |
| Mt2W | P2W | 0.014 | 0.0133 |
| OxC | P2W | 0.0128 | 0.0123 |
| SVW | P2W | 0.0123 | 0.0119 |
| SVC | P2W | 0.0088 | 0.0088 |
| SCMC | P2W | 0.0152 | 0.0146 |
| SJRN | PC | 0.0073 | 0.0071 |
| SJRW | PC | 0.0084 | 0.0082 |
| MtC | PC | 0.0159 | 0.015 |
| MiC | PC | 0.016 | 0.0153 |
| CuW | PC | 0.0071 | 0.007 |
| P1W | PC | 0.014 | 0.0134 |
| IxW | PC | 0.0158 | 0.015 |
| P2W | PC | 0.0139 | 0.0132 |
| PC | PC | 0.6873 | 0.0236 |
| P4W | PC | 0.0144 | 0.0138 |
| P5W | PC | 0.0139 | 0.0133 |
| OxW | PC | 0.0093 | 0.0089 |
| CarC | PC | 0.0134 | 0.0129 |
| Mt1W | PC | 0.0139 | 0.0133 |
| Mt2W | PC | 0.0138 | 0.0133 |
| OxC | PC | 0.0128 | 0.0124 |
| SVW | PC | 0.0124 | 0.0119 |
| SVC | PC | 0.0089 | 0.0087 |
| SCMC | PC | 0.0152 | 0.0145 |
| SJRN | P4W | 0.0071 | 0.0071 |
| SJRW | P4W | 0.0086 | 0.0084 |
| MtC | P4W | 0.016 | 0.0152 |
| MiC | P4W | 0.0158 | 0.015 |
| CuW | P4W | 0.0071 | 0.007 |
| P1W | P4W | 0.014 | 0.0134 |
| IxW | P4W | 0.0158 | 0.015 |
| P2W | P4W | 0.0138 | 0.0133 |
| PC | P4W | 0.0146 | 0.0139 |
| P4W | P4W | 0.7104 | 0.0229 |
| P5W | P4W | 0.0138 | 0.0132 |
| OxW | P4W | 0.0091 | 0.0089 |
| CarC | P4W | 0.0134 | 0.0129 |
| Mt1W | P4W | 0.0138 | 0.0132 |
| Mt2W | P4W | 0.0138 | 0.0132 |
| OxC | P4W | 0.0127 | 0.0123 |
| SVW | P4W | 0.0123 | 0.012 |
| SVC | P4W | 0.0092 | 0.009 |
| SCMC | P4W | 0.0152 | 0.0146 |
| SJRN | P5W | 0.007 | 0.0068 |
| SJRW | P5W | 0.0086 | 0.0084 |
| MtC | P5W | 0.0159 | 0.0151 |
| MiC | P5W | 0.016 | 0.0152 |
| CuW | P5W | 0.007 | 0.0069 |
| P1W | P5W | 0.0139 | 0.0133 |
| IxW | P5W | 0.0158 | 0.0151 |
| P2W | P5W | 0.0139 | 0.0133 |
| PC | P5W | 0.0144 | 0.0138 |
| P4W | P5W | 0.0144 | 0.0138 |
| P5W | P5W | 0.6868 | 0.021 |
| OxW | P5W | 0.0092 | 0.009 |
| CarC | P5W | 0.0133 | 0.0128 |
| Mt1W | P5W | 0.0138 | 0.0133 |
| Mt2W | P5W | 0.0137 | 0.0131 |
| OxC | P5W | 0.0128 | 0.0124 |
| SVW | P5W | 0.0123 | 0.0119 |
| SVC | P5W | 0.0088 | 0.0087 |
| SCMC | P5W | 0.0151 | 0.0145 |
| SJRN | OxW | 0.0072 | 0.0071 |
| SJRW | OxW | 0.0084 | 0.0082 |
| MtC | OxW | 0.0165 | 0.0159 |
| MiC | OxW | 0.0158 | 0.015 |
| CuW | OxW | 0.0071 | 0.0069 |
| P1W | OxW | 0.0138 | 0.0133 |
| IxW | OxW | 0.0163 | 0.0156 |
| P2W | OxW | 0.014 | 0.0133 |
| PC | OxW | 0.0145 | 0.0139 |
| P4W | OxW | 0.0145 | 0.0139 |
| P5W | OxW | 0.0138 | 0.0132 |
| OxW | OxW | 0.7567 | 0.033 |
| CarC | OxW | 0.0134 | 0.0129 |
| Mt1W | OxW | 0.0138 | 0.0132 |
| Mt2W | OxW | 0.0139 | 0.0133 |
| OxC | OxW | 0.0129 | 0.0125 |
| SVW | OxW | 0.0123 | 0.0117 |
| SVC | OxW | 0.0092 | 0.0088 |
| SCMC | OxW | 0.0152 | 0.0145 |
| SJRN | CarC | 0.0072 | 0.007 |
| SJRW | CarC | 0.0086 | 0.0083 |
| MtC | CarC | 0.0159 | 0.0152 |
| MiC | CarC | 0.0158 | 0.0152 |
| CuW | CarC | 0.0069 | 0.007 |
| P1W | CarC | 0.0139 | 0.0133 |
| IxW | CarC | 0.016 | 0.0153 |
| P2W | CarC | 0.014 | 0.0133 |
| PC | CarC | 0.0145 | 0.0138 |
| P4W | CarC | 0.0146 | 0.0139 |
| P5W | CarC | 0.0139 | 0.0132 |
| OxW | CarC | 0.0094 | 0.009 |
| CarC | CarC | 0.6831 | 0.0188 |
| Mt1W | CarC | 0.0139 | 0.0132 |
| Mt2W | CarC | 0.0139 | 0.0133 |
| OxC | CarC | 0.0129 | 0.0123 |
| SVW | CarC | 0.0123 | 0.0119 |
| SVC | CarC | 0.0091 | 0.0089 |
| SCMC | CarC | 0.015 | 0.0144 |
| SJRN | Mt1W | 0.0072 | 0.007 |
| SJRW | Mt1W | 0.0086 | 0.0085 |
| MtC | Mt1W | 0.0157 | 0.0151 |
| MiC | Mt1W | 0.0159 | 0.0151 |
| CuW | Mt1W | 0.0071 | 0.0068 |
| P1W | Mt1W | 0.0138 | 0.0133 |
| IxW | Mt1W | 0.0158 | 0.0151 |
| P2W | Mt1W | 0.014 | 0.0133 |
| PC | Mt1W | 0.0144 | 0.0138 |
| P4W | Mt1W | 0.0144 | 0.0139 |
| P5W | Mt1W | 0.014 | 0.0135 |
| OxW | Mt1W | 0.0094 | 0.0091 |
| CarC | Mt1W | 0.0133 | 0.0128 |
| Mt1W | Mt1W | 0.686 | 0.0235 |
| Mt2W | Mt1W | 0.014 | 0.0134 |
| OxC | Mt1W | 0.0128 | 0.0124 |
| SVW | Mt1W | 0.0122 | 0.0118 |
| SVC | Mt1W | 0.009 | 0.0088 |
| SCMC | Mt1W | 0.0151 | 0.0145 |
| SJRN | Mt2W | 0.007 | 0.0068 |
| SJRW | Mt2W | 0.0086 | 0.0082 |
| MtC | Mt2W | 0.0159 | 0.0152 |
| MiC | Mt2W | 0.0159 | 0.0151 |
| CuW | Mt2W | 0.007 | 0.007 |
| P1W | Mt2W | 0.014 | 0.0135 |
| IxW | Mt2W | 0.0159 | 0.0152 |
| P2W | Mt2W | 0.014 | 0.0134 |
| PC | Mt2W | 0.0146 | 0.0139 |
| P4W | Mt2W | 0.0145 | 0.0139 |
| P5W | Mt2W | 0.0139 | 0.0133 |
| OxW | Mt2W | 0.0093 | 0.009 |
| CarC | Mt2W | 0.0132 | 0.0127 |
| Mt1W | Mt2W | 0.014 | 0.0134 |
| Mt2W | Mt2W | 0.6865 | 0.0245 |
| OxC | Mt2W | 0.0128 | 0.0124 |
| SVW | Mt2W | 0.0122 | 0.0118 |
| SVC | Mt2W | 0.0091 | 0.0088 |
| SCMC | Mt2W | 0.0149 | 0.0143 |
| SJRN | OxC | 0.0069 | 0.0068 |
| SJRW | OxC | 0.0086 | 0.0086 |
| MtC | OxC | 0.0158 | 0.0151 |
| MiC | OxC | 0.0158 | 0.0152 |
| CuW | OxC | 0.0073 | 0.0071 |
| P1W | OxC | 0.0137 | 0.0132 |
| IxW | OxC | 0.0159 | 0.0151 |
| P2W | OxC | 0.014 | 0.0135 |
| PC | OxC | 0.0146 | 0.0139 |
| P4W | OxC | 0.0145 | 0.0139 |
| P5W | OxC | 0.014 | 0.0133 |
| OxW | OxC | 0.0092 | 0.009 |
| CarC | OxC | 0.0133 | 0.0127 |
| Mt1W | OxC | 0.0138 | 0.0133 |
| Mt2W | OxC | 0.0138 | 0.0133 |
| OxC | OxC | 0.7223 | 0.0275 |
| SVW | OxC | 0.0124 | 0.012 |
| SVC | OxC | 0.009 | 0.0088 |
| SCMC | OxC | 0.0151 | 0.0143 |
| SJRN | SVW | 0.0072 | 0.007 |
| SJRW | SVW | 0.0085 | 0.0083 |
| MtC | SVW | 0.016 | 0.0153 |
| MiC | SVW | 0.0158 | 0.015 |
| CuW | SVW | 0.0072 | 0.0071 |
| P1W | SVW | 0.0139 | 0.0133 |
| IxW | SVW | 0.0159 | 0.0151 |
| P2W | SVW | 0.0139 | 0.0133 |
| PC | SVW | 0.0146 | 0.0139 |
| P4W | SVW | 0.0146 | 0.014 |
| P5W | SVW | 0.0139 | 0.0133 |
| OxW | SVW | 0.0092 | 0.0091 |
| CarC | SVW | 0.0135 | 0.0129 |
| Mt1W | SVW | 0.014 | 0.0134 |
| Mt2W | SVW | 0.014 | 0.0133 |
| OxC | SVW | 0.0129 | 0.0124 |
| SVW | SVW | 0.6919 | 0.0346 |
| SVC | SVW | 0.009 | 0.0088 |
| SCMC | SVW | 0.0152 | 0.0145 |
| SJRN | SVC | 0.0072 | 0.0068 |
| SJRW | SVC | 0.0087 | 0.0084 |
| MtC | SVC | 0.0167 | 0.0158 |
| MiC | SVC | 0.0158 | 0.0151 |
| CuW | SVC | 0.007 | 0.0069 |
| P1W | SVC | 0.0137 | 0.0131 |
| IxW | SVC | 0.016 | 0.0153 |
| P2W | SVC | 0.0139 | 0.0131 |
| PC | SVC | 0.066 | 0.032 |
| P4W | SVC | 0.0144 | 0.0138 |
| P5W | SVC | 0.014 | 0.0134 |
| OxW | SVC | 0.0092 | 0.009 |
| CarC | SVC | 0.0646 | 0.0265 |
| Mt1W | SVC | 0.0394 | 0.0227 |
| Mt2W | SVC | 0.0396 | 0.0226 |
| OxC | SVC | 0.0597 | 0.0279 |
| SVW | SVC | 0.0986 | 0.0421 |
| SVC | SVC | 0.8302 | 0.0274 |
| SCMC | SVC | 0.0599 | 0.0272 |
| SJRN | SCMC | 0.0073 | 0.0069 |
| SJRW | SCMC | 0.0086 | 0.0083 |
| MtC | SCMC | 0.0159 | 0.0151 |
| MiC | SCMC | 0.016 | 0.0152 |
| CuW | SCMC | 0.0071 | 0.0068 |
| P1W | SCMC | 0.0139 | 0.0133 |
| IxW | SCMC | 0.0159 | 0.015 |
| P2W | SCMC | 0.014 | 0.0133 |
| PC | SCMC | 0.0146 | 0.014 |
| P4W | SCMC | 0.0146 | 0.014 |
| P5W | SCMC | 0.014 | 0.0134 |
| OxW | SCMC | 0.0093 | 0.0089 |
| CarC | SCMC | 0.0134 | 0.013 |
| Mt1W | SCMC | 0.0139 | 0.0134 |
| Mt2W | SCMC | 0.0139 | 0.0133 |
| OxC | SCMC | 0.0128 | 0.0125 |
| SVW | SCMC | 0.0124 | 0.012 |
| SVC | SCMC | 0.0089 | 0.0086 |
| SCMC | SCMC | 0.6828 | 0.0162 |

**S6 Table.** Annotation of outliers with possible signs of selection with Gene Ontology in *Agave potatorum.*

| ***ID*** | ***GO_ID*** | ***Function*** | ***GO Description*** |
| --- | --- | --- | --- |
| loc4623_pos166 | GO:0008168 | Molecular Function | Methyltransferase activity |
| loc6160_pos134 | GO:0006468 | Biological Process | Protein phosphorylation |
| loc6160_pos134 | GO:0004672 | Molecular Function | Protein kinase activity |
| loc6160_pos134 | GO:0005524 | Molecular Function | ATP binding |
| loc7553_pos29 | GO:0071949 | Molecular Function | FAD binding |
| loc7553_pos29 | GO:0050660 | Molecular Function | FAD binding |
| loc9095_pos7 | GO:0005515 | Molecular Function | Protein binding |
| loc9150_pos9 | GO:0008270 | Molecular Function | Zinc ion binding |
| loc9150_pos9 | GO:0016491 | Molecular Function | Oxidoreductase activity |
| loc12451_pos216 | GO:0005515 | Molecular Function | Protein binding |
| loc13651_pos12 | GO:0003676 | Molecular Function | Nucleic acid binding |
| loc14782_pos481 | GO:0006468 | Biological Process | Protein phosphorylation |
| loc14782_pos481 | GO:0048544 | Biological Process | Recognition of pollen |
| loc14782_pos481 | GO:0004672 | Molecular Function | Protein kinase activity |
| loc14782_pos481 | GO:0005524 | Molecular Function | ATP binding |
| loc14782_pos481 | GO:0004674 | Molecular Function | Serine/threonine kinase activity |
| loc15614_pos383 | GO:0006633 | Biological Process | Fatty acid biosynthetic process |
| loc15614_pos383 | GO:0016746 | Molecular Function | Acyltransferase activity |
| loc15614_pos383 | GO:0016747 | Molecular Function | Acyltransferase activity |
| loc15614_pos383 | GO:0016020 | Cellular Component | Component membrane |
| loc22265_pos31 | GO:0006468 | Biological Process | Protein phosphorylation |
| loc22265_pos31 | GO:0005515 | Molecular Function | Protein binding |
| loc22265_pos31 | GO:0004672 | Molecular Function | Protein kinase activity |
| loc22265_pos31 | GO:0005524 | Molecular Function | ATP binding |
| loc25521_pos530 | GO:0016788 | Molecular Function | Hydrolase activity |
| loc25575_pos278 | GO:0006629 | Biological Process | Lipid metabolic process |
| loc29338_pos22 | GO:0008270 | Molecular Function | Zinc ion binding |
| loc31384_pos94 | GO:0006468 | Biological Process | Protein phosphorylation |
| loc31384_pos94 | GO:0004672 | Molecular Function | Protein kinase activity |
| loc31384_pos94 | GO:0005515 | Molecular Function | Protein binding |
| loc31384_pos94 | GO:0005524 | Molecular Function | ATP binding |
| loc37128_pos104 | GO:0008270 | Molecular Function | Zinc ion binding |

**S1 Fig.** Diversity index. a) expected heterozygosity; b) observed heterozygosity; c) Inbreeding index (*F_IS_*) per locality in *Agave potatorum*, estimated with 14,875 SNPs.

**S2 Fig.** Kinship analysis (relatedness phi) in *Agave potatorum*.

******

**S3 Fig.** Genetic difference. a) Genetic distance (*F_ST_*) per locality; b) UPGMA per individual in *Agave potatorum*, estimated with 14,875 SNPs.

******

**S4 Fig.** Mantel test, showing the correlation between genetic distance (*F_ST_*) and geographic distance in the populations of *Agave potatorum*.

******

**S5 Fig.** Outliers identified with BayeScan software with *BH*= 0.05 and *pvalue* of 0.01 in *Agave potatorum.*

******
